# *QuickStats*: Percentage* of Children Having a Problem for Which Prescription Medication Has Been Taken Regularly for ≥3 Months,^†^ by Age Group and Sex — National Health Interview Survey, United States, 2017^§^

**DOI:** 10.15585/mmwr.mm6744a9

**Published:** 2018-11-09

**Figure:**
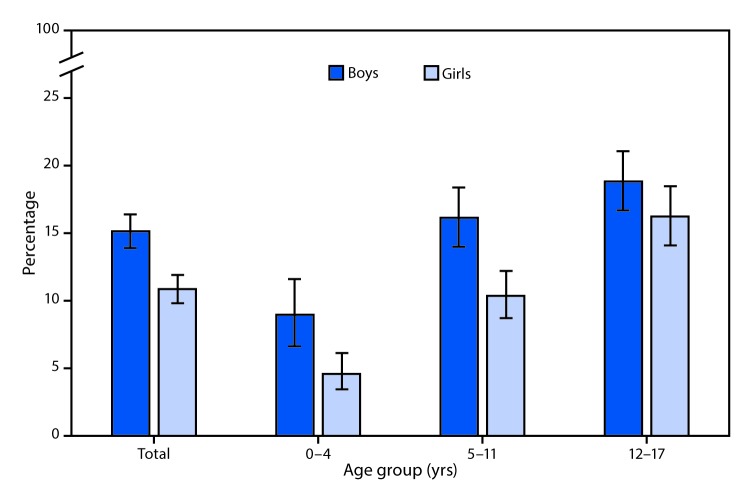
In 2017, the percentage of children who had a problem for which prescription medication had been taken regularly for ≥3 months increased with increasing age. Among boys the percentage ranged from approximately 8% of those aged 0–4 years to nearly 19% of those aged 12–17. Among girls the percentage ranged from approximately 5% of those aged 0–4 years to 16% of those aged 12–17. Overall, boys were more likely than girls to have had a problem for which prescription medication had been taken regularly for ≥3 months.

Source: National Health Interview Survey, 2017 data. https://www.cdc.gov/nchs/nhis.htm.

